# Foliar fertilizer and irrigation effects on mung bean: implications for leaf physiology and yield

**DOI:** 10.3389/fpls.2025.1704065

**Published:** 2025-10-29

**Authors:** Mengjie Han, Renqiang Chen, Tongguo Gao, Huiyan Gao, Xiaoling Wang, Meng Liu, Tiebing Lv, Baoliang Zhu, Huike Duan, Hongquan Liu

**Affiliations:** ^1^ College of Urban and Rural Construction, Hebei Agricultural University, Baoding, Hebei, China; ^2^ State Key Laboratory of North China Crop Improvement and Regulation, Hebei Agricultural University, Hebei, China; ^3^ College of Life Sciences, Hebei Agricultural University, Baoding, Hebei, China; ^4^ College of Horticulture, Hebei Agricultural University, Baoding, Hebei, China

**Keywords:** Mung bean, Foliar fertilizer, leaf traits, Photosynthetic characteristics, yield

## Abstract

Addressing the growing demand for food in semi-arid regions requires effective water and fertilizer strategies. An irrigation and foliar fertilization field experiment was performed in Baoding, Hebei Province, to determine the effects of foliar nutrition and water conditions on mung bean (*Vigna radiata* L.), with focus on leaf traits, photosynthetic characteristics, and yield. The treatments were irrigated at branching and flowering-podding stages. Foliar treatments were freshwater only, high-potassium, high-phosphorus, balanced fertilizer. The two-factor experiment comprised a total of eight treatments and twenty-four plots which were arranged randomly. Numerous parameters, including leaf traits, photosynthetic parameters, SPAD dynamics, and yield components, were determined. Results showed that under full irrigation, the high- phosphorus treatment (W1F2) significantly enhanced leaf area, net photosynthetic rate (*Pn*), and SPAD values by 18.1%, 14.4%, and 24.0%, respectively, at 7 days after spraying (DAS7), and delayed leaf senescence. Under water stress, the balanced fertilizer (W0F3) most effectively mitigated stress effects, showing the smallest reduction in leaf area and a 9.8% higher SPAD retention. Photosynthetic performance varied: W1F2 maintained the highest water use efficiency over 21 days under full irrigation, while W0F3 showed the least decline in Pn under water stress. Yield increased significantly with high- phosphorus treatment (F2), by 30.86% (under W1 conditions) and 37.79% (under W0 conditions), primarily due to a 20.66% and 19.17% rise in grains per pod, respectively. The W0F3 treatment also increased 100-seed weight by 8.12%, supporting yield advantage. In conclusion, high- phosphorus foliar fertilizer is recommended under sufficient irrigation to boost photosynthesis and sink strength, whereas the balanced fertilizer provides yield advantages under water-limited conditions by maintaining photosynthetic area and grain weight. This study provides a theoretical and practical foundation for precision foliar nutrient management in mung beans during the flowering and podding stages in semi-arid regions.

## 1 Introduction

Mung bean is an important edible legume crop widely cultivated in China due to its short growth cycle, stress tolerance, and high nutritional value (Hou et al., 2019). However, during the critical flowering and podding stage, plants often experience intense internal nutrient competition and various environmental stresses. These factors lead to premature leaf senescence, reduced photosynthetic capacity, and ultimately constrain yield potential (Yang et al., 2020).

Water is a key environmental factor governing crop photosynthesis and nutrient uptake (Bista et al., 2018). In semi-arid and semi-humid regions with uneven precipitation and frequent seasonal droughts, water stress often induces premature leaf senescence in mung beans and significantly impairs root nutrient acquisition from the soil (Islam et al., 2024). Photosynthesis constitutes the physiological foundation for dry matter accumulation and yield formation (Zhu et al., 2010; Ambavaram et al., 2014). During the pod-filling stage, sustaining high leaf photosynthetic capacity is crucial for allocating assimilates to the developing pods (Du et al., 2024). Water stress not only triggers stomatal closure, thereby reducing the net photosynthetic rate, but also disrupts cellular metabolism, ultimately limiting photoassimilate supply to developing sinks (Chaves et al., 2009; Flexas et al., 2009).

Under water stress, traditional soil-applied fertilizers often exhibit low nutrient use efficiency due to soil nutrient fixation and reduced root absorption capacity, making it difficult to meet crop demands in a timely manner (Wang et al., 2017). In contrast, foliar fertilization can bypass soil and root limitations, featuring rapid nutrient uptake and high utilization efficiency. It is therefore regarded as an effective approach for targeted nutrient supplementation and stress mitigation under water-limited conditions (Hadavi and Garcia-Mina, 2023). Nitrogen (N), phosphorus (P), and potassium (K) are essential macronutrients that play distinct roles in leaf growth and photosynthetic physiology, and they respond differently to water conditions (Lu et al., 2025). Specifically, P is integral to energy metabolism and the formation of membrane systems (White and Hammond, 2008). K, critical for drought resistance, functions primarily in regulating stomatal movement and maintaining cellular osmotic potential (Zörb et al., 2014). Meanwhile, N serves as the fundamental building block for chlorophyll and protein synthesis (Berger et al., 2020). In summary, this systematic investigation into the physiological impacts of different foliar fertilizers under varying water regimes provides critical insights into the mechanisms of water-nutrient interactions, offering a scientific basis for precision management in mung bean cultivation.

While numerous studies have examined the individual effects of either water management or foliar fertilization on crop growth, a systematic investigation that simultaneously links irrigation regimes with foliar fertilizer types is still lacking. Such a study is needed to comprehensively assess their interactive effects on leaf architecture, photosynthetic function, and yield formation throughout the entire pod-setting cycle of mung bean. Determining the optimal foliar fertilizer under specific water conditions is crucial for developing precise management strategies; however, research in this area remains limited.

This study established two irrigation regimes: well-watered and water-stressed. At the flowering and podding stage, mung bean plants were treated with one of three foliar fertilizers-high-phosphorus, high-potassium, or balanced-which were applied at an equivalent rate but with distinct nutrient ratios; a water-sprayed group served as the control. The primary objective was not to establish a dose-response relationship but to conduct a preliminary screening by systematically comparing the physiological effects and temporal dynamics of these fertilizer types under different water conditions. This study focused on the flowering and podding stage, a period characterized by intense source–sink competition that critically determines yield. Through continuous dynamic monitoring, we systematically analyzed the temporal effects of water–fertilizer interactions on mung bean leaf area, dry matter accumulation, SPAD values, photosynthetic parameters, and yield components. The objectives were to (1) elucidate the physiological responses of different foliar fertilizers under varying water availability, and (2) decipher the synergistic mechanisms by which water–fertilizer interactions delay leaf senescence and enhance yield. The findings provide a foundation for subsequent in-depth studies and for screening effective foliar fertilizer strategies under different water regimes. It is important to note that this was a single-year, single-site controlled experiment. The conclusions primarily reveal the underlying physiological mechanisms in a specific context, and their practical applicability requires further validation across diverse years and ecological regions.

## 2 Materials and methods

### 2.1 Experimental site and materials

**Figure 1:**
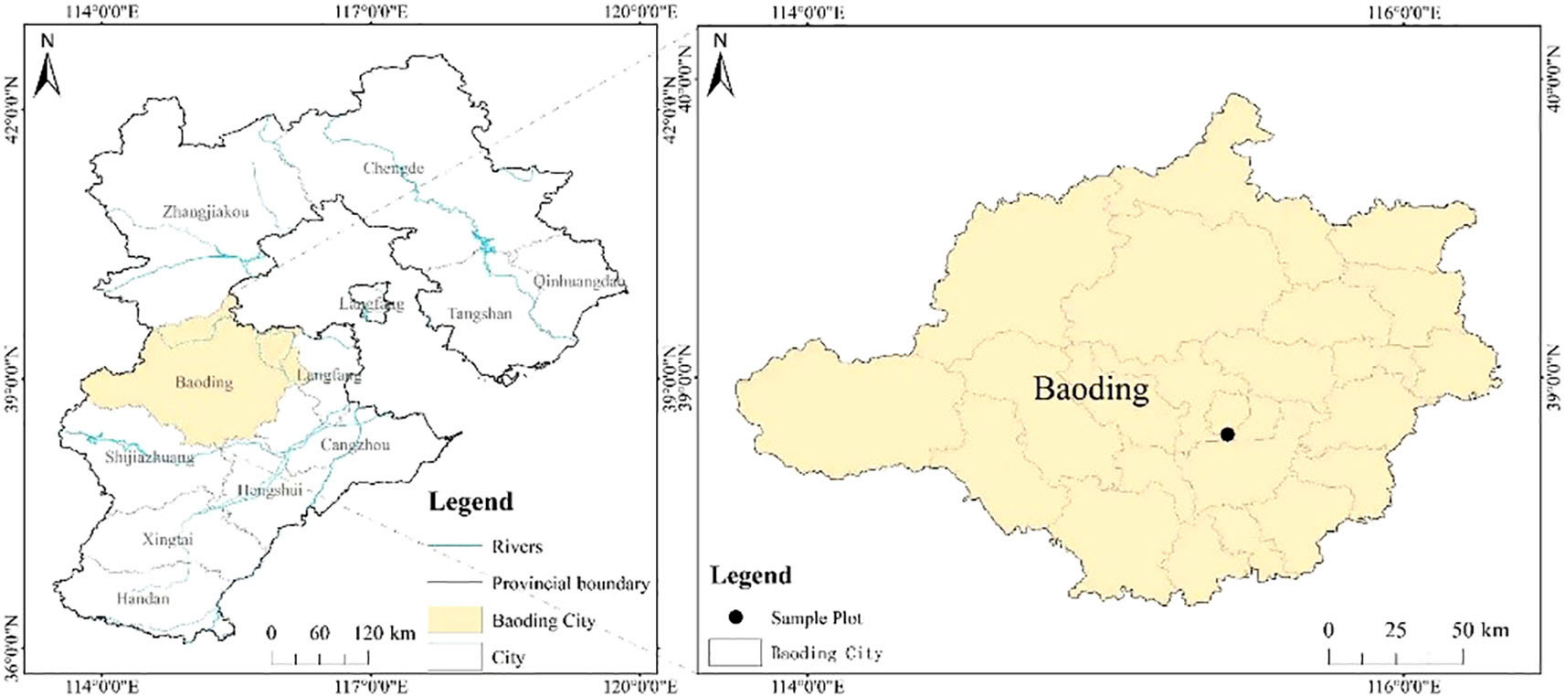
Research Area Location Map.

**Figure 2:**
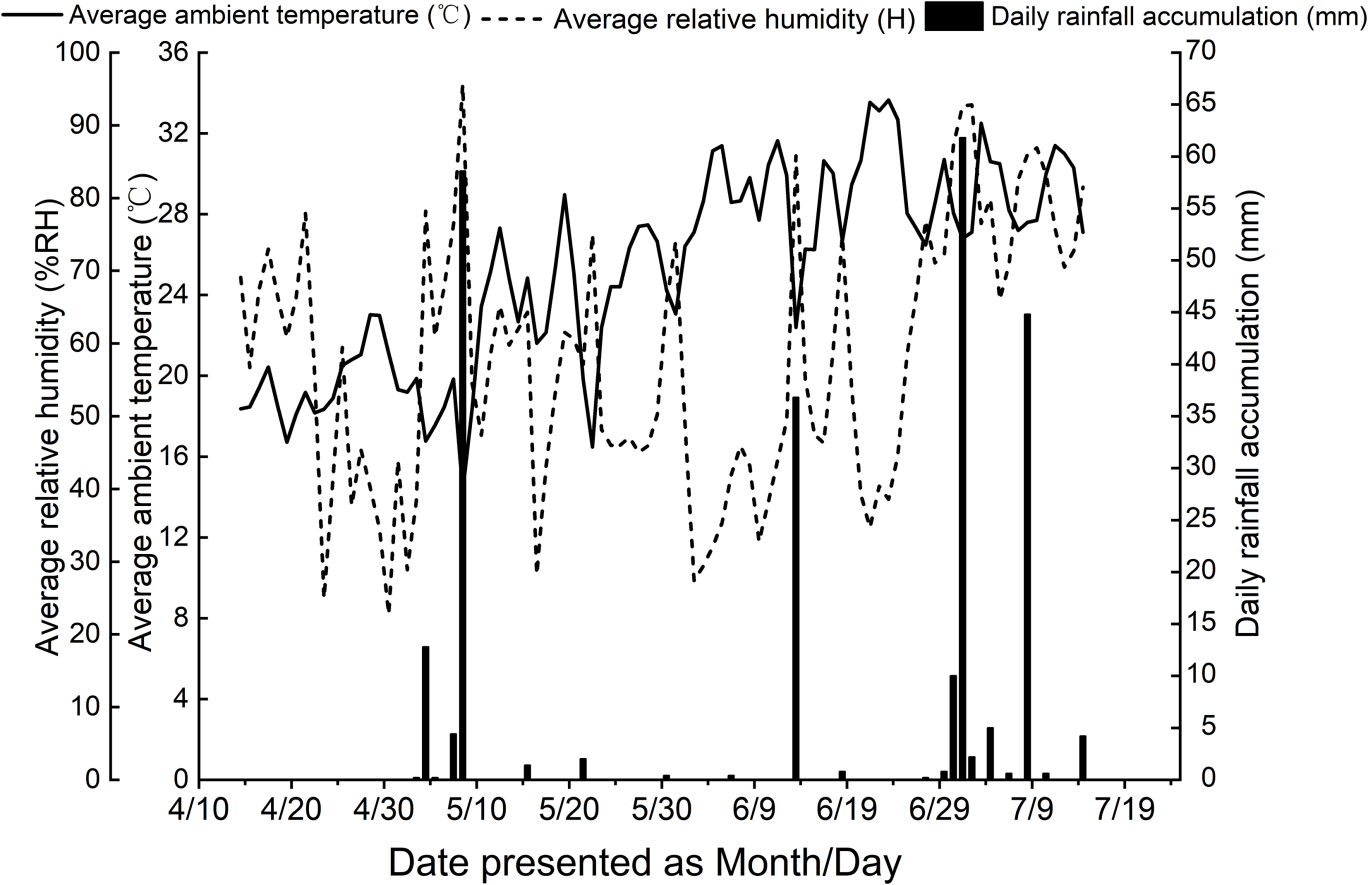
Changes in main meteorological elements during the growth period of mung bean (2025).

The field experiment was conducted in 2025 at the Agricultural Science Teaching Experimental Base of He Bei Agricultural University, located in Baoding City (38°81′N, 115°40′E, 21.7 m altitude; see **Figure 1**). This region has a warm temperate continental monsoon climate, characterized by semi-arid to semi-humid conditions. The mean annual temperature and precipitation are 13.4 °C and 498.9 mm, respectively, with the majority of rainfall occurring from June to August. The frost-free period typically lasts 165–210 days, and the annual evaporation averages 1430.5 mm. The experimental field featured loam soil with uniform fertility and a level topography. Key chemical properties of the top 30 cm plough layer are presented in **Table 1**. Meteorological data recorded during the experimental period were provided in **Figure 2**.

**Table 1:** Selected chemical characteristics of the surface soil (0–30 cm).

pH	Organic matter (g kg^-1^)	Total N (g kg^-1^)	Effective P (mg kg^-1^)	Quick-acting K (mg kg^-1^)
7.8	21.8	1.1	18.6	98.5

### 2.2 Experimental design and field management

The mung bean cultivar used was ‘Jilv 24’.

The field experiment was conducted from sowing on April 26 to harvest on July 17, 2025. Prior to sowing, a base fertilizer was applied uniformly at a rate of 450 kg ha^-^¹, using a compound fertilizer with an N-P_2_O_5_-K_2_O ratio of 15-15-15. Mung beans were sown in hills with a row spacing of 45 cm and a plant spacing of 20 cm, surrounded by a 40 cm buffer zone. The experiment employed a randomized complete block design with three replications. It included two irrigation regimes: W0 (irrigated once at the branching stage) and W1 (irrigated once each at the branching and flowering-podding stages). These were combined with four foliar spray treatments: F0 (freshwater only), F1 (high-potassium fertilizer), F2 (high-phosphorus fertilizer), and F3 (balanced fertilizer). This resulted in a total of eight treatment combinations. The 24 experimental plots (8 treatments × 3 replications) each measured 10 m^2^.

Foliar fertilizers were applied during the flowering and podding stages of mung bean growth. These developmental stages were defined as follows: the flowering stage began when 50% of plants had opened their first flower, and the podding stage when 50% of plants had developed young pods measuring 1–2 cm in length. Applications were made on calm, sunny evenings, once at the flowering stage (June 14) and again at the peak podding stage (June 21). The spray volume was 0.2 g·m^-2^, prepared at a concentration of 1 g·L^-1^. The concentration and application rate for all foliar fertilizers were determined based on preliminary trials and local agronomic practices, enabling a direct comparison of nutrient formulation effects under consistent application conditions. Freshly prepared solutions were sprayed evenly using a hand sprayer until leaves were fully wetted but not dripping. All other field management practices were uniform across treatments.

The commercial and laboratory-developed foliar fertilizers used in this study contained supplementary micronutrients (e.g., Zn, Mn, B) or humic acid in addition to nitrogen, phosphorus, and potassium (**Table 2**). These composite formulations reflect commercially available products and are intended to represent real-world agricultural practices. The core objective of this experiment was to evaluate the overall effect of these fertilizer types as complete functional units under different water regimes, rather than to isolate the individual contributions of NPK and the additional components.

**Table 2:** Formulation and composition of the foliar fertilizers.

Foliar fertilizer composition	Content	Foliar fertilizers were obtained from
High-K foliar fertilizer	NPK ratio: 10-5-40; Total micronutrients (Zn+Mn+B): ≤ 0.5%.	Hebei Mengbang Water Soluble Fertilizer Co., Ltd.
High-P foliar fertilizer	NPK: 10-46-10; Humic acid: ≤ 6%; Micronutrients (Zn+Mn+B+others): ≤ 3%	Laboratory-formulated, College of Life Sciences, Hebei Agricultural University
Balanced fertilizer	NPK: 20-20-20; Humic acid: ≤ 4.2%.	Laboratory-formulated, College of Life Sciences, Hebei Agricultural University

The composition of the foliar fertilizers is presented in **Table 2**.

### 2.3 Measurement items and methods

Measurements of all physiological indicators were focused on the flowering and podding stage, spanning 21 days after the foliar spray, to capture the complete dynamics from onset to decline within this key window.

Leaf trait measurement: Fully expanded leaves at the 3rd to 4th node of the main stem were sampled from non-border plants in each plot. Leaf length, width, and area were measured immediately. The leaves were then oven-dried at 105 °C for 30 minutes, followed by drying at 80 °C to constant weight to determine leaf dry weight.

Two key functional traits were calculated:

Leaf mass per area (*LMA*, g·m^-2^) = Leaf dry weight (mg)/Leaf area (cm^2^).
Leaf length-to-width ratio = Leaf length/leaf width.

Measurement of Photosynthetic Parameters: Photosynthetic parameters were measured at 7, 14, and 21 days after foliar fertilizer application. Between 9:00 and 11:00 AM on each sampling day, three fully expanded compound leaves were selected per plot. Using an LCi-SD portable photosynthesis system, the following parameters were recorded: net photosynthetic rate (*Pn*, μmol·m^-2^·s^-1^), transpiration rate (*Tr*, mmol m^-2^·s^-1^), stomatal conductance (*Gs*, mmol·m^-2^·s^-1^), and intercellular CO_2_ concentration (*Ci*, μmol·mol^-1^). Leaf-level water use efficiency (*WUE*) was calculated as the ratio of *Pn* to *Tr* (mmol CO_2_·mol^-^¹ H_2_O).

To ensure comparability, all measurements were conducted under stable and suitable environmental conditions. Key environmental factors-photosynthetically active radiation (*PAR*), atmospheric CO_2_ concentration (*Ca*), and leaf temperature (*Tleaf*)-were recorded simultaneously during each measurement. The conditions during the experiment were maintained as follows:

*PAR*: 1000-1500 μmol·m^-2^·s^-1^ (Saturating light intensity).
*Ca*: 380-420 μmol·mol^-1^.
*Tleaf*: 28-32 °C.

Yield and yield components: Prior to harvest, yield components were determined from two randomly selected 1 m^2^ areas per plot. Within each area, the number of plants and pods were counted to calculate pods per plant. Additionally, the number of seeds per pod was determined from a subsample of pods. At harvest, all plants within the designated areas were threshed to determine grain yield. The 100-seed weight was measured from a random sample of seeds after drying to constant weight.

### 2.4 Data processing

Crop experimental data were recorded and processed using Microsoft Excel 2019. Statistical analyses were performed with SPSS 26.0 software. Treatment effects were evaluated by one-way ANOVA followed by least significant difference (*LSD*) *post hoc* tests for multiple comparisons, with statistical significance set at *P* < 0.05. All data in figures and tables are presented as mean ± standard deviation. The sample size (*n*) for each measurement is explicitly stated throughout the manuscript and corresponds to the following biological replicates:

Leaf traits and SPAD values: fixed marker plants per plot (n = 3).
Photosynthetic parameters: three sampled plants per plot (n = 3).
Yield components: two 1 m² quadrats per plot (n = 2).
Plot yield and 100-seed weight: values from each replicate plot (n = 3).
Figures were prepared using Origin 2024.

## 3 Results

### 3.1 Leaf traits of Mung bean in response to foliar fertilizer and irrigation

**Figure 3:**
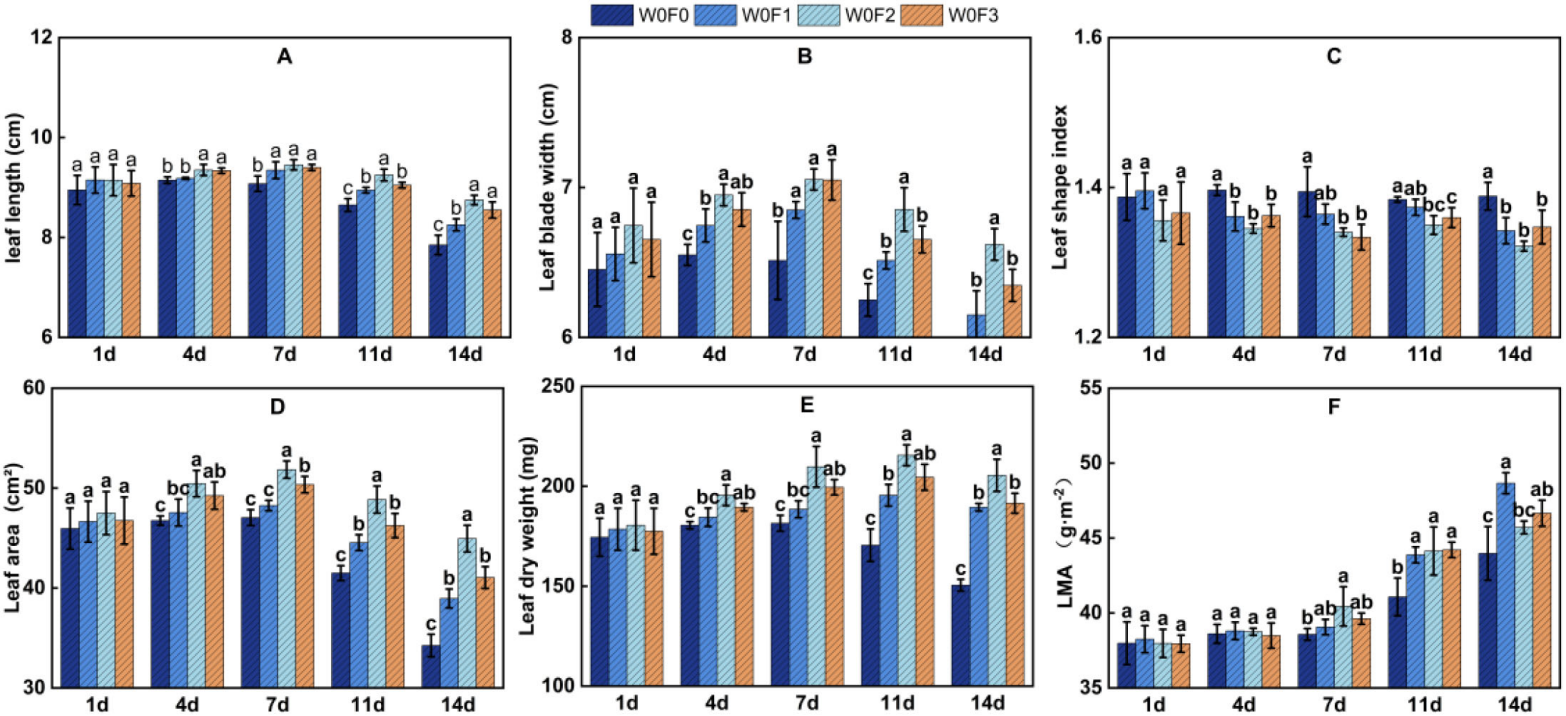
Effects of foliar fertilizer on leaf trait dynamics in mung bean under water stress during flowering and podding stages. **(A)** Leaf length, **(B)** Leaf width, **(C)** Leaf shape index, **(D)** Leaf area, **(E)** Leaf dry weight, **(F)** Leaf mass per area (*LMA*). Different lowercase letters indicate significant differences among treatments at *P* < 0.05. The same notation applies to the following figures.

**Figure 4:**
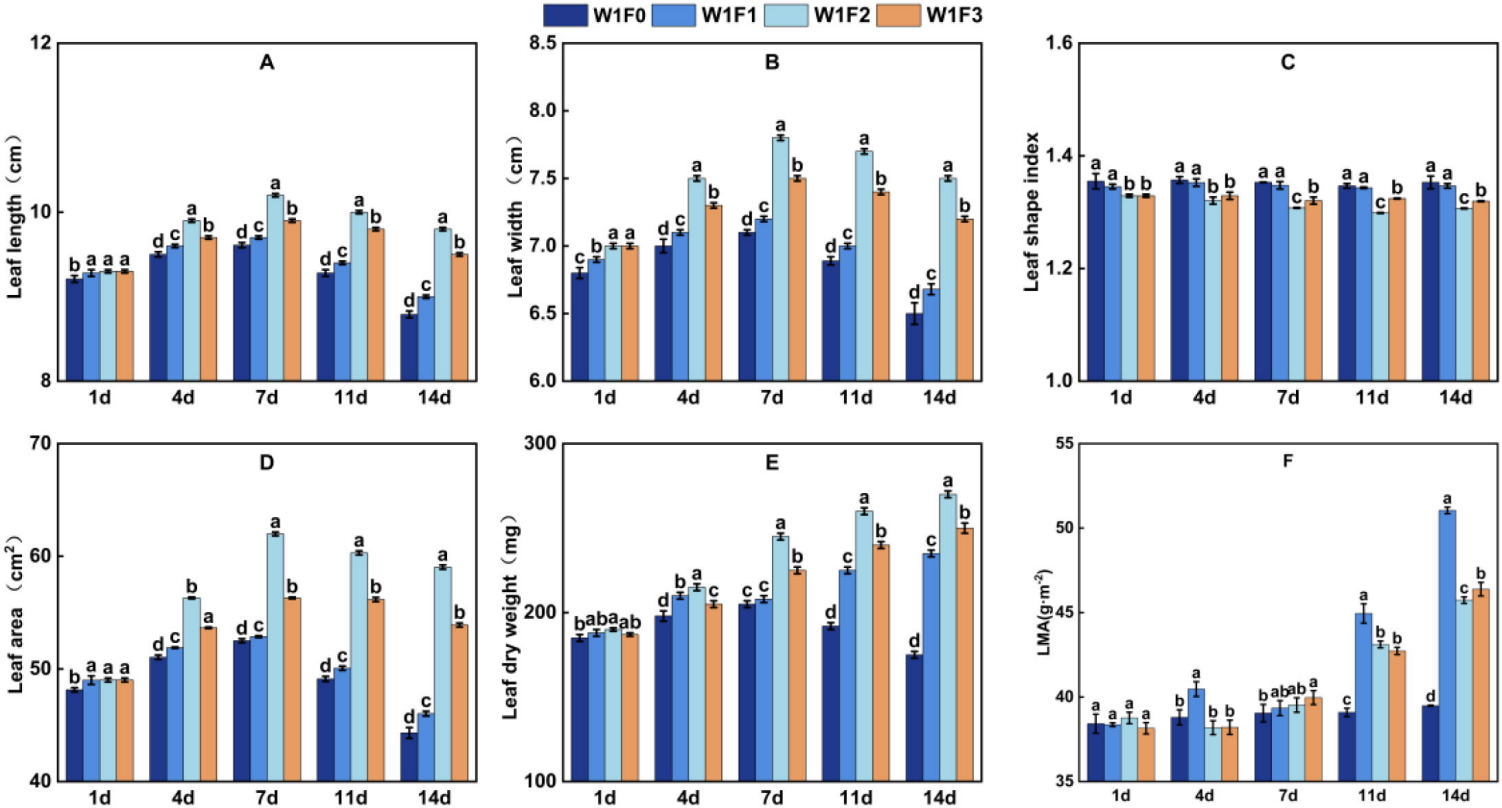
Effects of foliar fertilizer on leaf trait dynamics in mung bean under sufficient irrigation during flowering and podding stages. **(A)** Leaf length, **(B)** leaf width, **(C)** leaf shape index, **(D)** leaf area, **(E)** leaf dry weight, **(F)** leaf mass per area (*LMA*). The lowercase letters indicate significant differences among treatments at P < 0.05.

As shown in **Figures 3**, **4**, and **Table 3**, foliar fertilizer type and irrigation regime interacted significantly to influence mung bean leaf traits at the five sampling time points (DAS 1, 4, 7, 11, and 14), with effects varying dynamically over time. Under sufficient irrigation, leaf development was generally promoted. Furthermore, foliar fertilizer application, particularly the high-phosphorus treatment, significantly delayed the leaf senescence process during the later experimental stage.

**Table 3:** Fitted quadratic model parameters for SPAD dynamics of mung bean as influenced by foliar fertilizer and irrigation during flowering and podding stages.

Treatment	Peak SPAD Value	Fitting equation
W0F0	39.06	y=-0.009x^2^-0.612x+42.18(R^2^ = 0.99) ^**^
W0F1	42.28	y=-0.029x^2^-0.222x+41.86(R^2^ = 0.99) ^**^
W0F2	41.70	y=-0.036x^2^+0.148x+41.55(R^2^ = 0.99) ^**^
W0F3	43.33	y=-0.02x^2^-0.342x+41.87(R^2^ = 0.99) ^**^
W1F0	42.96	y=-0.025x^2^+-0.159x+42.2(R^2^ = 0.99) ^**^
W1F1	43.32	y=-0.066x^2^+0.724x+41.33(R^2^ = 0.94) ^**^
W1F2	44.53	y=-0.062x^2^+0.916x+41.15(R^2^ = 0.82) ^**^
W1F3	42.46	y=-0.056x^2^+0.436x+41.61(R^2^ = 0.97) ^**^

** indicates the statistical significance of the quadratic regression model at the P < 0.01 level.

No significant differences in leaf traits were observed among treatments on the first day after spraying, confirming consistent initial conditions. Treatment effects became apparent by day 4. Under well-watered conditions (W1), all foliar fertilizer treatments exhibited significantly greater leaf area and leaf dry weight compared to the W1F0 control (*p* < 0.05). The W1F2treatment showed the most substantial enhancement, with leaf area and dry weight increasing significantly by 10.4% and 9.1%, respectively. At this stage, the W1F1 treatment displayed the highest specific leaf weight (*LMA*), suggesting early promotion of biomass accumulation per unit leaf area. Under water-limited conditions (W0), foliar fertilizer treatments also outperformed the W0F0 control, though the effects were generally less pronounced than under W1. Furthermore, W1F2 exhibited a significantly lower leaf shape index than W1F0, indicating a tendency to develop broader, shorter leaves through enhanced horizontal expansion.

By day 7, the treatment effects had become more pronounced. Under well-watered conditions (W1), the W1F2 treatment reached peak values in both leaf area and leaf dry weight, showing significant increases of 18.1% and 20.4%, respectively, compared to the W1F0 control, followed by the W1F3 treatment. Although W1F2 exhibited the largest leaf area, its relatively low *LMA* indicated a leaf development strategy prioritizing rapid expansion. In contrast, W1F3 displayed the highest *LMA*, suggesting a “high-investment” strategy that favored dry matter accumulation per unit leaf area. Under water-limited conditions (W0), W0F2 still demonstrated the best performance in promoting leaf area, with a further reduction in leaf shape index indicating more pronounced development of shorter, broader leaves.

Between days 11 and 14, leaves in the F0 treatment exhibited clear signs of senescence, whereas those treated with foliar fertilizer retained their functional integrity. By day 14, the leaf area in the W1F0 treatment had decreased by 15.6% compared to that on day 7. In contrast, the reduction in leaf area was significantly less pronounced in all foliar fertilizer treatments. Among them, W1F2 maintained leaf area and leaf dry weight values that were 33.2% and 54.3% higher, respectively, than those of W1F0, demonstrating the most pronounced senescence-delaying effect. Compared to the control, leaf dry weight increased by 54.3%, 42.9%, and 34.3% for W1F2, W1F3, and W1F1, respectively. The advantage of W1F1 in biomass accumulation became particularly evident at this stage, with its *LMA* reaching 5.11 g·m^-^², significantly exceeding other treatments. This indicates that high-potassium foliar fertilizer uniquely enhanced dry matter deposition into leaves during later growth stages.

Irrigation regime significantly influenced mung bean leaf traits. Overall, leaf area, leaf dry weight, and *LMA* under well-watered (W1) conditions were consistently higher than those under water-limited (W0) conditions across all treatments. The high-phosphorus treatment was the most responsive to water availability, exhibiting more pronounced advantages under W1 than under W0. *LMA* was generally elevated under water stress, suggesting that mung bean tends to develop thicker leaves as a conservative growth strategy in moisture-deficient environments. Under W0 conditions, both high-phosphorus and balanced foliar fertilizers maintained relatively favorable leaf traits, indicating their superior adaptability to water stress.

### 3.2 SPAD values of Mung bean in response to foliar fertilizer and irrigation

**Figure 5:**
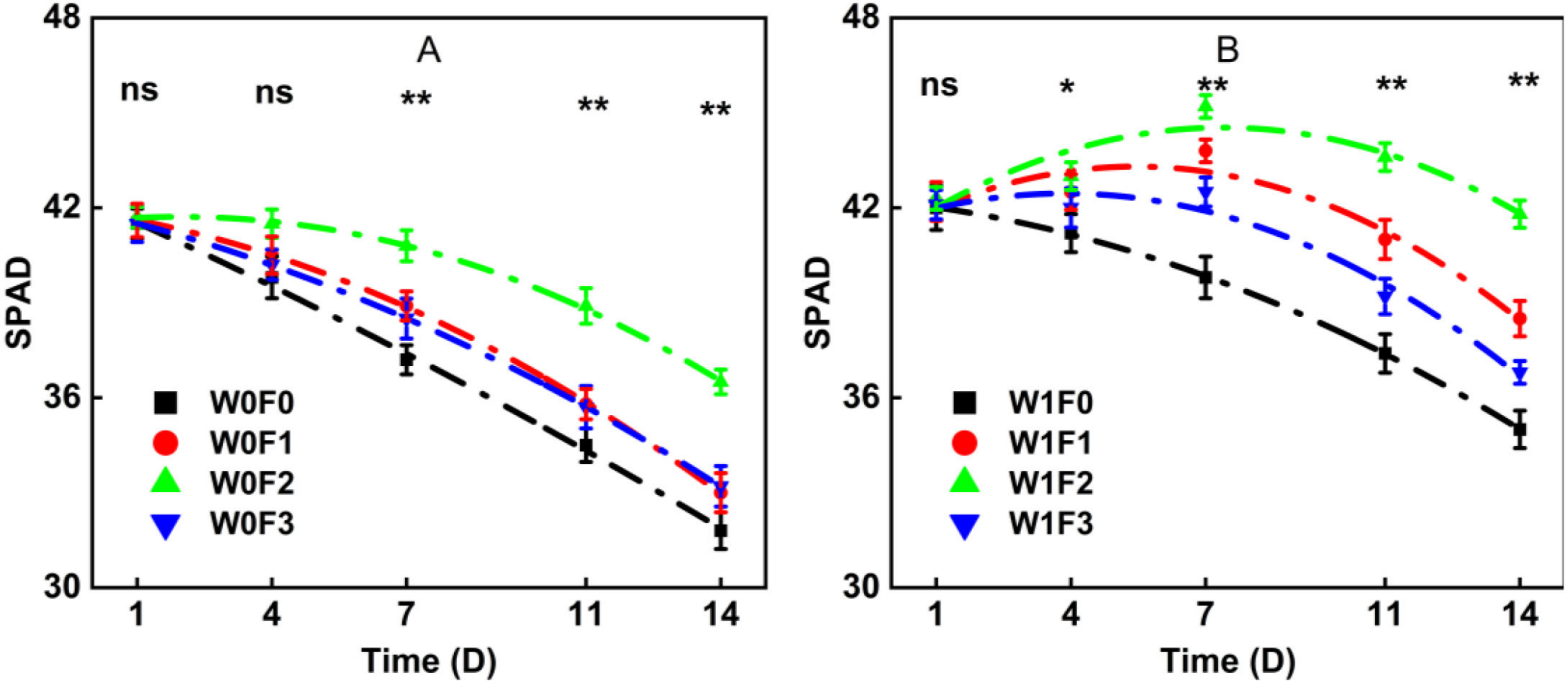
Effects of foliar fertilizer on SPAD dynamics in mung bean during flowering and podding stages under **(A)** water stress and **(B)** full irrigation. ns means no significant difference (P > 0.05); * and ** means significant difference at P < 0.05 level or at P < 0.01 level, respectively.

As depicted in **Figure 5**, the dynamics of SPAD values in all treatments followed a quadratic function (y = ax² + bx + c, a < 0), exhibiting a characteristic unimodal pattern that reflects the natural progression of leaf development followed by senescence. The significantly negative quadratic coefficient for each treatment (**Table 3**) confirms that SPAD values began to decline rapidly after peaking. Furthermore, both irrigation and foliar fertilizer application significantly influenced the characteristic parameters of this senescence process.

Under W1 irrigation, the W1F2treatment achieved the highest peak SPAD value of 44.68, which also occurred the latest (DAS7.39) and closely aligned with the flowering and podding period of mung bean. This indicates that high-phosphorus foliar fertilizer effectively prolonged the functional leaf photosynthesis duration under adequate moisture. In contrast, the SPAD peaks of both the W1F0and W1F3 treatments appeared before the early monitoring period, followed by a continuous decline. Although the W1F1 treatment peaked during the critical growth stage (5.48 days), its maximum value and persistence were lower than those of W1F2.During the 0–7 day ascending phase, the SPAD value of W1F2 continued to increase, rising by 1.78 units between days 4 and 7, whereas other treatments began to decline within the same period, highlighting the mid- to late-stage advantage of the high-phosphorus treatment. In the subsequent decline phase (7–14 days), W1F2 showed the slowest reduction, while W1F0 and W1F3 decreased by 4.97 and 5.70, respectively. By day 14, the SPAD value of W1F2 remained 19.98% higher than that of W1F0, and this advantage continued to widen as growth progressed.

**Figure 6:**
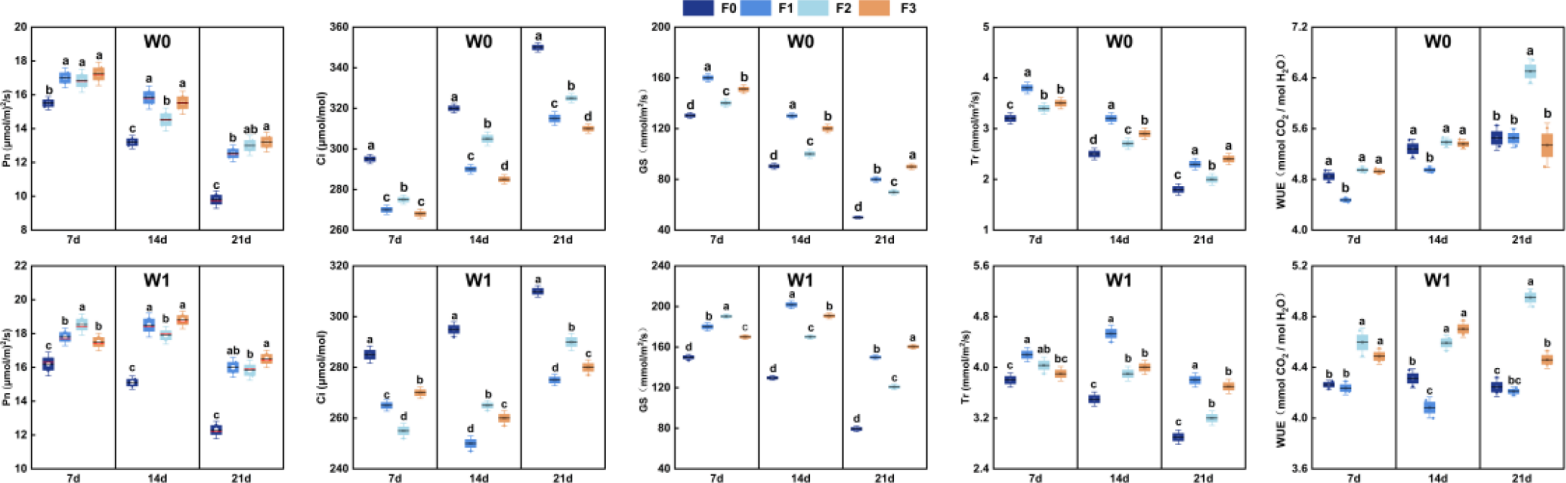
Photosynthetic parameters of mung bean as influenced by foliar fertilizer under well-watered (W1) and water-stressed (W0) conditions during flowering and podding stages. (Parameters include: net photosynthetic rate, *Pn*; intercellular CO_2_ concentration, *Ci*; transpiration rate, *Tr*; stomatal conductance, Gs; water use efficiency, *WUE*.).

Under W0 (water-limited) conditions, the SPAD dynamics of all treatments exhibited distinct trends (**Figure 6A**). The W0F2 treatment reached a lower peak value of 43.15, which also occurred earlier. Nevertheless, it remained significantly later than the W0F0 control, indicating that high-phosphorus fertilization still delayed chlorophyll degradation under water stress. The W0F1 treatment showed relatively stable SPAD values throughout the observation period, with minimal fluctuation, underscoring the role of potassium in maintaining chlorophyll stability under drought conditions. In contrast, the W0F3 treatment performed better during the later stage (11–14 days), with only a 2.50 decrease in SPAD, compared to a 2.70 decline in W0F0 over the same period. This suggests that the balanced foliar fertilizer helped delay leaf senescence in a water-deficient environment.

Regarding the ability to maintain SPAD values throughout the flowering and podding period, the treatments under well-watered (W1) conditions ranked in the order W1F2 > W1F1 > W1F3 > W1F0, with W1F2 being significantly higher than all others at every time point. Under water-deficient (W0) conditions, the ranking was W0F2 > W0F3 > W0F1 > W0F0. The relative advantage of W0F2 was particularly pronounced during the later stage, exhibiting a 14.78% higher value than W0F0 on day 14.

On day 4, the SPAD advantage of the high-phosphorus treatment paralleled increases in both leaf area and leaf dry weight, suggesting the early establishment of a synergistic mechanism that enhanced both photosynthetic performance and biomass accumulation. By day 14, the retention rates of SPAD and dry matter (relative to day 7) were highly consistent, reinforcing the comprehensive advantage of the high-phosphorus treatment in delaying leaf senescence. The quadratic model provided an excellent fit to the SPAD dynamics under all treatments, with coefficients of determination (*R* ^2^) ranging from 0.82 to 0.99 across water conditions. The R² values for F0 and F1 reached 0.99, demonstrating the model’s high accuracy in capturing temporal SPAD trends.

In summary, both foliar fertilizer type and irrigation regime strongly influenced SPAD values in mung bean leaves. Under sufficient water, the high-phosphorus fertilizer delayed chlorophyll degradation and extended the period of high photosynthetic efficiency, thereby enhancing the sustained supply of photo assimilates. Under water stress, the balanced fertilizer offered superior chlorophyll retention, while the high-potassium treatment provided a stable protective effect. These findings establish a theoretical basis for selecting context-specific foliar fertilization strategies to optimize mung bean performance under varying water availability.

### 3.3 Mung bean photosynthesis in response to foliar fertilizer and irrigation

Photosynthetic parameters were measured at 7, 14, and 21 days after foliar fertilizer application during the flowering and podding stage (**Figure 6**). Under well-watered conditions (W1), W1F0 showed a continuous decline in *Pn*, *Gs*, and *Tr* as leaves senesced, while *Ci* gradually increased, reflecting typical aging-related patterns. In contrast, foliar fertilizer treatments overall effectively mitigated this decline, exhibiting a dynamic response characterized by rapid initiation, sustained peak activity, and delayed decrease in photosynthetic function.

Foliar fertilizer application significantly boosted photosynthetic performance by the 7th day. Moreover, photosynthetic parameters under W1 conditions were superior to those under W0 conditions across all treatments (**Figure 6**). Under W1, W1F2 showed the strongest stimulation of *Pn* and *Gs*, reaching 18.53 μmol·m^-2^·s^-1^-an increase of 14.38% in *Pn* and 26.89% in *Gs* over W1F0. Concurrently, its *Ci* decreased significantly by 10.5%, reflecting high CO_2_ assimilation efficiency. W1F1 exhibited the highest Tr, exceeding the W1F0 by 10.53%, whereas W1F2 achieved 7.98% greater *WUE* than W1F0. Under W0 conditions, although absolute values of all photosynthetic parameters were lower, trends remained consistent: W0F2 maintained the highest *Pn* and *Gs* (8.58% and 7.69% above W0F0, respectively), and W0F1 retained higher Tr (9.38% increase over W0F0).

By 21 days after spraying, the role of foliar fertilizers in delaying photosynthetic decline became more pronounced. Under well-watered (W1) conditions, the photosynthetic advantage of the W1F3treatment expanded further, with *Pn* increasing by 34.15% compared to W1F0-significantly higher than both W1F1 and W1F2. Both W1F3 and W1F1 maintained substantially higher *Gs*, exceeding the control by 102.07% and 89.08%, respectively. Ultimately, W1F2 still showed significantly higher *WUE* than W1F0, underscoring the ability of phosphorus-enriched fertilizer to stabilize and optimize water use in later stages. Under water-limited (W0) conditions, W0F3 achieved the highest *Pn*, 34.69% above the W0F0, while W0F1 maintained the strongest *Gs*, exceeding W0F0 by 60.00%. Notably, W0F2 exhibited the most pronounced *WUE* advantage, with a significant 19.26% increase over the control, whereas W0F1 showed no significant difference in *WUE* from the W0F0.

In summary, foliar fertilizer and irrigation exhibited significant interactive effects on photosynthetic characteristics of mung bean during the flowering and podding stages. The high-phosphorus fertilizer was instrumental in achieving rapid photosynthetic initiation and sustaining optimal water use efficiency throughout the growth cycle. In the mid to late stages, the balanced and high-potassium fertilizers were more effective at maintaining photosynthetic activity and delaying senescence. Notably, the high-potassium formulation played a critical role in preserving stomatal function under water-limited conditions.

### 3.4 Mung bean yield and yield components as influenced by foliar fertilizer and irrigation

As shown in **Table 4**, all three foliar fertilizer treatments significantly increased mung bean yield under both irrigation regimes, with clear differences among treatments. The yield enhancement was more pronounced under well-watered (W1) conditions. Although yields under water stress (W0) were lower than those under W1, they remained significantly higher than the control under the corresponding irrigation conditions.

**Table 4:** Mung bean yield and yield components as influenced by foliar fertilizer and irrigation.

Treatment	pods per plant	100-seed weight (g)	Seeds per pod	Yield (kg ha^-^¹)
W0F0	20.86 ± 0.49d	7.39 ± 0.29d	7.51 ± 0.32c	1591.39 ± 80.13d
W0F1	21.07 ± 0.37d	7.75 ± 0.37cd	7.99 ± 0.53abc	1879.06 ± 127.58c
W0F2	22.15 ± 0.51bcd	7.67 ± 0.19ab	8.95 ± 0.21bc	2192.85 ± 75.39b
W0F3	21.37 ± 0.31cd	7.99 ± 0.2bc	8.41 ± 0.19ab	2071.67 ± 119.56b
W1F0	22.69 ± 1.01abc	7.68 ± 0.21cd	7.89 ± 0.42bc	1931.57 ± 24.09c
W1F1	23.09 ± 0.92ab	7.95 ± 0.19bc	8.42 ± 0.38ab	2173.00 ± 39.93b
W1F2	23.78 ± 1.23a	7.85 ± 0.19a	9.52 ± 0.34ab	2527.58 ± 19.72a
W1F3	23.47 ± 1.23ab	8.18 ± 0.16b	8.78 ± 0.51a	2397.18 ± 24.4a
ANOVA (F)				
W	30.64*	7.91*	4.87*	96.34**
F	2.23ns	17.44**	5.80**	63.13**
W×F	0.09ns	0.08ns	0.08ns	0.10ns

Data represent mean ± SD (*n* = 3). Different lowercase letters within a column indicate significant differences among foliar fertilizer treatments under the same irrigation regime at *P* < 0.05 according to *LSD* test. Results of two-way ANOVA show main effects of irrigation (W) and foliar fertilizer (F), and their interaction (W × F). Significance levels: *P < 0.05, **P < 0.01, ns, not significant.

Regarding yield components, the W1F2 treatment under well-watered (W1) conditions produced the most seeds per pod, exceeding W1F0 by 20.66%, while theW1F3 and W1F1 treatments showed increases of 11.28% and 6.72%, respectively. Under water-limited (W0) conditions, W0F2 maintained the highest seeds per pod, with a 19.17% increase over W0F0, compared to 11.98% and 6.39% for W0F3 and W0F1, respectively. For 100- seed weight, the W1F3 treatment under W1 conditions reached the highest value (8.18 g), showing no significant difference from W1F1 and W1F2 but being significantly higher than W1F0. Under W0 conditions, the advantage of W0F3 was more pronounced, with a significant 8.12% increase in 100- seed weight compared to W0F0, while W0F1 and W0F2 showed no significant difference from W0F0.

Pod number per plant did not differ significantly between foliar fertilizer treatments and the control under the same irrigation regime, indicating that yield gains were primarily achieved through enhanced seeds per pod and grain weight. Overall, mung bean yield across irrigation conditions consistently ranked F2 > F3 > F1 > F0. Under well-watered (W1) conditions, W1F2, W1F3, and W1F1 treatments showed yield increases of 30.86%, 24.11%, and 12.50%, respectively, compared to W1F0. Under water-limited (W0) conditions, yields for W0F2, W0F3, and W0F1 were 37.79%, 30.18%, and 18.07% higher, respectively, than W0F0.

## 4 Discussion

### 4.1 Physiological effects and synergistic mechanisms of different foliar fertilizers under regulated water conditions

This study revealed significant interactive effects of foliar fertilizer and irrigation on leaf traits, photosynthetic performance, and senescence dynamics in mung bean (**Tables 5**, **6**). The efficacy of different nutrient formulations depended on water availability, with each type exerting distinct physiological functions under specific moisture regimes. The two irrigation levels-well-watered and water-stressed-were established to simulate key field scenarios of flowering-stage drought. The results demonstrate that water conditions not only influenced the magnitude of foliar fertilizer effects but also shaped their underlying physiological mechanisms and senescence patterns.

**Table 5:** Results of the three-way ANOVA for the effects of water regime (W), foliar fertilization (F), and time (T) on mung bean leaf traits.

Treatment	Leaf length	Leaf width	Leaf area	Leaf dry weight	Leaf shape index	*LMA*	SPAD
W	660.40^**^	795.69^**^	1410.68^**^	849.53^**^	119.66^**^	1.37 ns	1395.96^**^
F	96.98^**^	178.82^**^	305.13^**^	280.94^**^	52.85^**^	79.61^**^	351.26^**^
T	159.16^**^	94.63^**^	207.44^**^	135.13^**^	5.10^**^	518.34^**^	839.98^**^
W×F	15.61^**^	6.66^**^	21.69^**^	11.39^**^	1.67 ns	12.43^**^	19.00^**^
W ×T	32.52^**^	17.07^**^	77.11^**^	48.06^**^	1.92 ns	3.53^*^	93.92^**^
F × T	8.96^**^	7.65^**^	25.61^**^	38.10^**^	1.03 ns	26.64^**^	34.38^**^
W×F×T	1.16ns	1.22 ns	4.01^**^	3.90^**^	1.04 ns	5.51^**^	2.72^**^

ns means no significant difference (*P*>0.05); * and ** means significant difference at *P* < 0.05 level or at *P* < 0.01 level, respectively.

**Table 6:** Photosynthetic characteristics of mung bean Three-factor analysis of variance results of different water (W), fertilization (F) and time (T) treatments on photosynthetic characteristics of mung bean.

Treatment	Pn	Gs	Ci	Tr	WUE
W	1020.27^**^	6889^**^	2189.17^**^	1683.91^**^	1091.31^**^
F	350.39^**^	1255.78^**^	716.55^**^	149.60^**^	104.93^**^
T	864.27^**^	3589.72^**^	1335.9^**^	565.03^**^	125.79^**^
W×F	7.73^**^	119.45^**^	29.79^**^	2.79ns	6.16^**^
W ×T	96.23^**^	344.19^**^	255.9^**^	99.62^**^	91.70^**^
F × T	21.88^**^	100.68^**^	28.98^**^	10.72^**^	23.87^**^
W×F×T	4.78^**^	46.06^**^	10.52^**^	3.37^**^	4.85^**^

ns means no significant difference (P > 0.05); ** means significant difference at P < 0.01 level.

Phosphorus plays a central role in triggering rapid physiological responses and supporting structural development in plants. In this study, the high-phosphorus foliar fertilizer induced the greatest increases in leaf area, leaf dry weight, and net photosynthetic rate (*Pn*) between 4 and 7 days after application (**Figures 3**, **6**), consistent with the multiple key functions of phosphorus in plant metabolism and growth. Phosphorus serves as a fundamental component of ATP and phospholipids, providing energy for cell division and expansion while forming the structural basis of membranes-collectively driving rapid leaf establishment (Cetner et al., 2020). These results align with findings in soybean, where foliar phosphorus application significantly enhanced leaf ATP content and net photosynthetic rate (Rodrigues et al., 2025). Furthermore, as an essential cofactor of Rubisco activase, phosphorus directly elevated carbon assimilation efficiency, accounting for the highest Pn and lowest *Ci* observed in high-phosphorus treated plants during early stages (Parry, 2003). This phosphorus-mediated photosynthetic priming effect has also been documented in other legumes such as kidney bean (Abdelrahman et al., 2018; Karpinska and Foyer, 2025), suggesting it represents a conserved physiological response to phosphorus nutrition in leguminous species.

Potassium plays a central role in stomatal regulation and the maintenance of physiological homeostasis. In contrast to the growth‐promoting strategy of phosphorus, potassium contributes primarily to the stable maintenance of plant functions. This study demonstrated that the high-potassium treatment exhibited the highest *LMA*, most stable SPAD values, and optimal *Gs* and *Tr* during the mid-to-late experimental period (days 7-14). These responses are attributed to the role of K^+^ as a key osmotic regulator, which maintains stomatal aperture by modulating guard cell turgor to ensure CO_2_ supply (Hu et al., 2021). Concurrently, potassium ions help stabilize chloroplast membrane structure and mitigate lipid peroxidation, thereby delaying functional leaf senescence (Johnson et al., 2022). Under water stress, the osmotic adjustment and stomatal protection conferred by potassium became particularly critical, enabling the high-K treatment to sustain relatively higher Gs and a more gradual SPAD decline in later stages, illustrating its contribution to yield stability under stress (Wang et al., 2013). These findings align with the view of Khatun et al. (2021) that potassium contributes to drought resistance in legume crops primarily through physiological regulation rather than direct structural roles (Khatun et al., 2021).

The balanced foliar fertilizer was characterized by its nutrient synergy and consistent performance throughout the growing cycle. Its effect was intermediate between the high-phosphorus and high-potassium treatments, with the most notable attribute being its sustained effectiveness, particularly during days 14-21. In terms of photosynthetic maintenance and delayed senescence, the balanced treatment consistently ranked among the highest in both *Pn* and *Gs*. This stability stems from its balanced nutrient formulation: nitrogen ensures continued synthesis of photosynthetic proteins and chlorophyll (Wang et al., 2021), phosphorus supports energy metabolism (Cetner et al., 2020), and potassium enhances physiological hhomeostasis (Johnson et al., 2022). This synergistic combination helps prevent limitations associated with any single nutrient under water stress, thereby conferring broad adaptability and stable yield performance across varying moisture regimes. It is important to note that excessive nitrogen application during the critical reproductive stage can stimulate excessive vegetative growth, leading to delayed maturity and impaired pod development (Zhang et al., 2017; Wang et al., 2023). Since the basal nitrogen application already satisfied the basic growth requirements of mung bean, this study did not include a separate high-nitrogen treatment. Instead, the balanced foliar fertilizer served as a reference to evaluate the synergistic effects of nitrogen, phosphorus, and potassium.

In summary, this study systematically evaluated the physiological effects of different nutritional strategies embodied by high-phosphorus, high-potassium, and balanced foliar fertilizers. It is pertinent to address the role of nitrogen and the corresponding experimental design. The consistent performance of the balanced formulation (F3) highlights the cornerstone role of nitrogen in maintaining leaf function in the mid-to-late stages. As a fundamental element for chlorophyll and protein synthesis, nitrogen explains the superior maintenance of SPAD values and the net photosynthetic rate (*Pn*) observed in the F3 treatment. The absence of an independent high-nitrogen treatment was a deliberate design choice, predicated on the established agronomic understanding of the flowering and podding stages: while nitrogen demand remains significant, its priority shifts from driving vegetative growth to ensuring a stable supply for reproductive development. An excessive nitrogen supply risks destabilizing the source-sink balance, thereby impairing the allocation of photoassimilates to the pods. Thus, supplying nitrogen in a balanced proportion with phosphorus and potassium (as in F3), or as an auxiliary component in the high-phosphorus and high-potassium formulations, was demonstrated to be a safer and more efficacious strategy. Future investigations should consider implementing nitrogen level gradients to accurately quantify the response curve of mung bean to foliar nitrogen application during the flowering and podding stages.

### 4.2 The interaction between water and fertilizer and optimization strategies

This study provides direct physiological evidence for the role of high-phosphorus foliar fertilizer in enhancing both source capacity and sink development. Regarding source strength, the W1F2 treatment exhibited the highest Pn and lowest *Ci* on DAS 7. The significantly reduced *Ci* indicated efficient CO_2_ assimilation in mesophyll cells, demonstrating that phosphorus fertilization directly enhanced the dark reaction processes. Concurrently, this treatment achieved the largest leaf area, highest SPAD peak, and slowest subsequent decline, consolidating source function across both physical scale and temporal duration. In terms of sink strength, W1F2 produced the most seeds per pod, suggesting that robust early-stage source activity supplied ample photoassimilates for pod development, while phosphorus-through its roles in ATP and nucleic acid synthesis-provided energy and genetic material for endosperm cell division, thereby translating source advantages into actual sink expansion (Ma et al., 2021). Thus, a coherent physiological pathway links the strong early signals observed here with final yield components, supporting the dual function of high-phosphorus foliar fertilizer in coordinately regulating source–sink relationships.

Thus, under well-watered conditions, water sustains cell turgor and acts synergistically with phosphorus to maximize the leaf area and dry weight advantages of the high-phosphorus foliar fertilizer treatment (**Figures 3**, **4**). Even under water stress, the high-phosphorus treatment maintained a relative advantage by preserving membrane stability and enhancing water use efficiency, underscoring the role of phosphorus in stress physiology (**Figure 6**).

In this study, the high-potassium treatment demonstrated effective maintenance of leaf function, though its contribution to yield enhancement was relatively limited. This outcome may be explained by several factors. First, the experimental soil contained 98.5 mg kg^-1^ of available potassium (**Table 1**), a level considered above average. As a result, plants likely acquired substantial potassium from the soil to meet basic physiological demands, implying that foliar potassium application served primarily to fine-tune physiological performance-particularly under water stress-rather than to alleviate severe deficiency. Second, the inherent source-sink relationship and nutrient partitioning pattern of the cultivar ‘Jilv 24’ may exhibit genotypic specificity. In summary, under the specific soil and varietal conditions of this experiment, potassium fertilization played a distinct role in sustaining leaf function. It is plausible that in potassium-deficient soils or with genotypes more responsive to potassium, the yield-enhancing effect of potassium fertilization-particularly through increased grain number-could be more pronounced.

Water conditions play a dual role in this system, acting both synergistically with fertilizers and as a selective filter that determines which physiological mechanisms dominate. Full irrigation amplifies the positive effects of foliar fertilizers, particularly enhancing the growth-promoting potential of high-phosphorus formulations and the function-preserving capacity of high-potassium ones. Under water stress, however, plant priorities shift from maximizing growth to ensuring survival and maintaining yield. In this context, high-phosphorus fertilizer emerges as a dominant mechanism by improving water use efficiency; high-potassium fertilizer contributes through stomatal and osmotic regulation; and the balanced formulation provides comprehensive stability. Thus, irrigation regime serves as a key environmental filter that selects the optimal nutrient strategy in a given moisture environment (Barka et al., 2025). In the mung bean system, phosphorus-enriched fertilizer is optimal under ample water, whereas a balanced formulation is preferable under water limitation. This conclusion differs from potassium-optimized strategies reported in chickpea (Sattar et al., 2020), a divergence that may reflect differences in genetic traits or growth period priorities between the two species.

### 4.3 Linking leaf physiology and photosynthesis to yield components in Mung bean

This study demonstrated that improvements in leaf traits and photosynthetic performance ultimately translated into enhanced yield and its components, with different foliar fertilizers employing distinct pathways to achieve these gains. The high-phosphorus foliar fertilizer primarily enhanced yield by increasing sink capacity. In this study, the high-phosphorus treatment achieved the highest yield under both water regimes, primarily by significantly increasing the number of seeds per pod (**Table 4**). This outcome was directly linked to its early-stage advantages in net photosynthetic rate and leaf area. Abundant photoassimilates, together with the role of phosphorus in facilitating sucrose transport and promoting endosperm cell division, effectively minimized grain abortion, thereby expanding sink capacity (Ma et al., 2021). This finding aligns with reports by Chen in other legumes, where phosphorus was shown to enhance grain filling (Chen et al., 2025). Thus, the high-phosphorus foliar fertilizer not only strengthened source activity but also played a dual role in promoting sink establishment.

The balanced foliar fertilizer enhanced yield primarily by promoting assimilate partitioning to grains and increasing individual grain weight. Although its total yield ranked below the high-phosphorus treatment, its yield-enhancing mechanism differed: while it induced a smaller increase in seeds per pod than the high-phosphorus fertilizer, it achieved significantly higher 100-seed weight than all other treatments. These results indicate that the advantage of the balanced fertilizer lies not in markedly increasing grain number, but in sustaining leaf photosynthetic function through integrated nutrient supply during late reproductive stages. On this basis, it ensured a continuous provision of substrates for starch and protein synthesis, supporting efficient assimilate transport and complete grain filling during the pod-filling period. This trait proved particularly valuable for maintaining yield stability under water-stressed conditions (Bhavya et al., 2024; Janusauskaite, 2025).

The yield improvement from the high-potassium relied primarily on the preservation and extension of photosynthetic source function, rather than directly enhancing sink capacity. Although the high-K treatment resulted in a modest yield increase and showed no significant difference in 100-seed weight compared to the control, its mechanism operated through delaying leaf senescence and prolonging the photosynthetic period, thereby increasing total assimilate supply (Lu et al., 2019). Because potassium exerts limited direct influence on key sink formation processes such as endosperm cell division, its main contribution lies in securing a stable and sustained flow of assimilates (Zhou et al., 2023).

In summary, this study demonstrates that under well-watered conditions, high-phosphorus foliar fertilizer effectively enhanced photosynthesis and sink capacity, leading to higher yield. Under water-limited environments, the balanced foliar fertilizer reliably supported stable yield by maintaining photosynthetic area and grain weight through coordinated nutrient supply. The high-potassium formulation primarily contributed by sustaining physiological function and improving stress resilience. Through systematic analysis of the temporal effects of water-fertilizer interactions on leaf area, enhancing SPAD values, photosynthetic parameters, and yield components, this work provides physiological evidence for selecting efficient foliar fertilizer types in mung bean. These findings offer practical insights for mung bean production in ecologically similar regions.

### 4.4 Study limitations and future research directions

This study systematically elucidated the effects of foliar fertilizer and irrigation on leaf physiology, photosynthesis, and yield formation in mung beans during the flowering and podding stages. Through well-designed field experiments, we established a physiological basis and practical management strategies for selecting foliar fertilizers under specific water regimes. However, the conclusions of this study should be interpreted in light of its limitations, which also highlight important directions for future research.

The generalizability of this study’s findings requires further validation. As this research was a controlled field experiment conducted at a single location (Baoding, Hebei) in a single year (2025), the results-including the specific 30.86% yield increase from high-phosphorus foliar fertilizer under full irrigation-were influenced by the particular meteorological conditions of that year (**Figure 2**) and the specific soil properties of the site (**Table 1**). Therefore, caution should be exercised when extrapolating these conclusions to other ecological regions with different climatic patterns and soil types. For instance, the efficacy and optimal formulation of the water-fertilizer interaction may differ in areas experiencing more severe drought or higher precipitation. Future research should evaluate the stability and reliability of these foliar fertilizer strategies through multi-year, multi-location trials to establish their broader applicability.

Secondly, the experimental design, with its limited water and fertilizer gradients, imposes constraints on building accurate predictive models. This study employed two water regimes (full irrigation and water stress) and a single foliar fertilizer dose, which was sufficient for qualitative comparisons and exploratory mechanism analysis. However, this setup cannot reveal the continuous response curves of the crop to varying water and fertilizer inputs. Consequently, it is unable to determine optimal irrigation thresholds or the most economical fertilizer concentration, and it fails to characterize crop responses under moderate water stress. To enable true precision agriculture, future studies should incorporate gradient designs for both soil moisture and fertilizer concentration. This will allow for the construction of a quantitative, decision-support model for water-fertilizer coupling.

Furthermore, the complex composition of the compound foliar fertilizers presents a challenge for attributing the observed effects solely to core nutrients. The fertilizers used in this study were complex formulations containing nitrogen, phosphorus, potassium, and additional components such as trace elements (e.g., Zn, Mn, B) and humic acid. Although the performance differences were primarily attributed to the varying NPK ratios, the potential synergistic or additive effects of the other components cannot be ruled out. This design, which mirrors commercial product application, inherently precludes the precise separation and quantification of the individual contributions of NPK versus other constituents. Therefore, the conclusions of this study should be interpreted as an evaluation of the overall effect of the complete functional unit (i.e., the compound formula) under different water regimes. Future research aiming to decipher the specific mechanisms should employ more detailed factorial designs, including treatments with and without trace components, to accurately dissect the role of each element in the water-fertilizer interaction.

In summary, this study lays the groundwork for strategic water and fertilizer management during the flowering and podding stages of mung bean by identifying effective formulations and providing a physiological explanation for their efficacy. Future research, building upon the effective high-phosphorus and balanced fertilizers identified here, should prioritize the following: (1) validating these findings across multiple years and locations to assess their stability; (2) establishing water and fertilizer gradients to enable quantitative modeling; and (3) dissecting the independent contributions of individual components within the compound formulas. These efforts are expected to culminate in a scientific and practical precision management system for water and fertilizer application in mung bean cultivation.

## 5 Conclusion

This study, based on observations during the flowering and podding stage of mung bean, systematically examined how foliar fertilizers and irrigation regimes affect leaf growth, photosynthetic characteristics, and yield. The results demonstrated that high−phosphorus foliar fertilizer most effectively promoted leaf expansion, delayed chlorophyll degradation, increased leaf area, and extended the photosynthetic functional period. Meanwhile, high−potassium foliar fertilizer was particularly effective in enhancing *LMA* and maintaining chlorophyll stability under water stress. Moreover, adequate irrigation significantly amplified the positive effects of all foliar fertilizers. Regarding photosynthetic performance, foliar fertilization significantly enhanced photosynthetic capacity in mung bean by improving gas-exchange parameters. The high-phosphorus treatment exhibited the most rapid response by day 7, whereas the balanced formulation maintained the highest photosynthetic rate at day 21, demonstrating stronger senescence resistance. Under water stress, the high-potassium treatment was most effective in sustaining stomatal conductance, while high-phosphorus fertilizer was most effective in improving water use efficiency (*WUE*). In terms of final yield, foliar fertilizers increased grain production mainly by raising seeds per pod and 100-seed weight, with no significant effect on pod number per plant. The high-phosphorus foliar fertilizer resulted in the highest yield, with a 30.86% increase over the W1F0 control under full irrigation and a 37.79% increase over W0F0 under water stress. The balanced fertilizer proved to be a reliable option for maintaining stable yield and high grain weight under water-limited conditions.

In summary, under the experimental conditions of this study (Baoding, Hebei, 2025), high-phosphorus foliar fertilizer is recommended under sufficient irrigation to rapidly enhance photosynthetic initiation and sink capacity. Under water-limited conditions, the balanced foliar fertilizer provides more stable yield advantages by maintaining photosynthetic area and grain weight through balanced nutrient supply. These conclusions, drawn from single-year data during a key growth period, offer valuable theoretical guidance and technical reference for precision foliar nutrient management of mung bean in semi-arid regions. The efficacy and stability of these approaches should be further validated through multi-year and multi-location trials.

Based on these results, a segmented fertilization strategy is proposed: applying high-phosphorus foliar fertilizer early to stimulate photosynthetic establishment, followed by high-potassium fertilizer in mid to late stages to maintain photosynthetic activity. Such an approach aligns more precisely with the physiological demands of mung bean during flowering and pod filling. Future studies should focus on determining the optimal economic application rates for these formulations, thereby refining the technical framework for high-efficiency mung bean cultivation.

## Data Availability

The original contributions presented in the study are included in the article/supplementary material. Further inquiries can be directed to the corresponding author/s.
